# A very rare case of bacteraemia in a 4-year-old girl with osteopetrosis with probable *Leuconostoc lactis* infection

**DOI:** 10.1099/acmi.0.000439

**Published:** 2023-10-13

**Authors:** Ali Azghar, Manal Azizi, Mohammed Lahmer, Elmostapha Benaissa, Yassine Ben Lahlou, Noufissa Benajiba, Mostafa Elouennass, Adil Maleb

**Affiliations:** ^1^​ Laboratory of Microbiology, Mohammed VI University Hospital/Faculty of Medicine and Pharmacy (University Mohammed the First), Oujda, Morocco; ^2^​ Laboratory of Bioresources, Biotechnology, Ethnopharmacology and Health (LBBES)/Research Team "Cell Biology and Pharmacology Applied to Health Sciences/Faculty of Medicine and Pharmacy (University Mohammed the First), Oujda, Morocco; ^3^​ Department of Pediatrics, Mohammed VI University Hospital/Faculty of Medicine and Pharmacy (University Mohammed the First), Oujda, Morocco; ^4^​ Department of Bacteriology, Mohammed V Teaching Military Hospital, Rabat, Morocco; ^5^​ Epidemiology and Bacterial Resistance Research Team/BIO-INOVA Centre, Faculty of Medicine and Pharmacy (University Mohammed V), Rabat, Morocco

**Keywords:** bacteraemia, case report, *Leuconostoc lactis*, Morocco, osteopetrosis

## Abstract

*
Leuconostoc lactis* (*LLac*) is a Gram-positive coccus of the family *Leuconostocaceae *. It can be found in a variety of vegetables and dairy products. *LLac* is an opportunistic pathogen with intrinsic resistance to vancomycin and teicoplanin. In this case report, we discuss a rare case of *LLac*-associated bacteraemia in a patient with osteopetrosis. A 4-year-old girl was admitted to the paediatric emergency department with acute fever without other signs. Blood culture revealed an infection with *LLac*. Using the streptococcus antibiogram, the isolate was resistant to vancomycin, teicoplanin, rifampicin and sulfamethoxazole-trimethoprim but sensitive to β-lactams, gentamicin, streptomycin, azithromycin, clarithromycin, lincomycin, clindamycin and erythromycin. The patient was treated with intravenous ceftriaxone and gentamicin, and subsequently with oral amoxicillin. After a favourable course, she was discharged from the hospital on the 10th day. The modes of transmission and physiopathology of *LLac* remain unknown. Factors associated with this infection include compromised immunity, previous antibiotic therapy especially with vancomycin, and application of a central venous catheter. In our patient, the risk factors for infection were pancytopenia and multiple transfusions used to treat bone marrow failure. The source of the bacteraemia could have been the cutaneous route, but it could also have been digestive due to the reservoir of the bacteria. *LLac* is known as an opportunistic bacterium. Further studies on its pathogenesis and other risk factors are needed to understand the true prevalence of this potentially fatal bacterium in compromised individuals, such as the case of our patient.

## Introduction

*Leuconostoc lactis* (*LLac*) belongs to Gram-positive cocci of the family *Leuconostocaceae* [1]. This bacterium grows easily on columbia blood agar, but it is difficult to identify because of its rarity [2]. *LLac* can be morphologically confused with *Enterococcus* sp. or *Streptococcus* sp. in clinical microbiology laboratories where bacterial identification is limited to classical biochemical techniques [2]. It is intrinsically resistant to vancomycin and teicoplanin [3]. *LLac* can be found in various vegetables and dairy products, and also milk and wine [3]. In humans, it has been isolated from vaginal and stool samples [4, 5]. The biological agents committee classifies *LLac* in risk group 1 [6]. This group defines agents that are unlikely to cause infectious disease in humans [6]. However, published cases of infection with this agent in immunocompromised individuals have confirmed that it can be an opportunistic pathogen in humans [7–12]. Most of the reported cases were bloodstream infections, ventriculitis, osteomyelitis, meningitis, pneumonia, endocarditis, pleural empyema or urinary tract infections [7–12]. Several factors may be associated with these infections such as immunosuppression, catheter use and prior antibiotic therapy with vancomycin [7–12]. In this report, we discuss a rare case of a child diagnosed with osteopetrosis who had *LLac* bacteraemia. To the best of our knowledge, this association is the first to be documented worldwide.

## Case report

Our patient was a 4-year-old girl treated in the paediatric department of the Mohammed VI University Hospital (Oujda, Morocco) since 2015 for osteopetrosis with bone marrow aplasia. Her treatment was mainly based on blood transfusion and an oral iron chelator (deferiprone, 30 mg kg^–1^ day^–1^ in three divided doses). She was admitted to the emergency department (13 March 2018; day 1) with 4 days of acute fever without other symptoms. Clinical examination found a conscious, pale and febrile child (39.4 °C). She had a psychomotor delay, and growth failure with craniofacial dysmorphism. Abdominal examination found a distension with splenomegaly reaching the umbilicus, and hepatomegaly with discrete abdominal collateral venous circulation.

Her white blood cell count was normal (7560 µl^–1^) including 2160 µ^–1^ neutrophils, 4390 µl^–1^ lymphocytes and 900 µl^–1^ monocytes. Our patient was anaemic (4 g d^l−1^) associated with thrombocytopenia (13000 µl^–1^) and higher ferritin levels (3262 µg l^–1^). C-reactive protein and lactate dehydrogenase were elevated (148 mg l^−1^ and 429 UI l^–1^, respectively) (day 1). A complete blood count is summarized in Table 1.

**Table 1.** Complete blood count parameters

Haematological and biochemical parameters	Value
White blood cell count	7560 µl^–1^
Neutrophils	2160 µl^–1^
Lymphocytes	4390 µl^–1^
Monocytes	900 µl^–1^
Haemoglobin	4 g dl^−1^
Thrombocytopenia	13000 µl^–1^
Hyperferritinaemia	3262 µg l^−1^
C-reactive protein	148 mg l^−1^
Lactate dehydrogenase	429 UI l^–1^
**Microbiological results**	
Presumptive identification	Aero-anaerobic, Gram-positive cocci, negative oxidase and catalase activities
Final identification (BD Phoenix NID Panel)	* Leuconostoc lactis* (99.99 % confidence)

Two blood culture aerobic bottles (BD Aerobic, BACTEC; Becton Dickinson), taken at a 7 h interval, were incubated in the BD BACTEC FX-400 (Becton Dickinson Microbiology Systems) automated blood culture system according to the manufacturer’s instructions (day 1). Immediately after the second blood sampling, a probabilistic antibiotic therapy combining ceftriaxone (50 mg kg^–1^ 24 h^–1^) and gentamicin (5 mg kg^–1^ 24 h^–1^) was started (day 1). No further sampling for a possible entry point to the bloodstream was performed. In addition, the time to positivity of both blood culture bottles was 2 h. Microscopic examination after Gram staining found abundant Gram-positive cocci in both bottles. Subculture of both bottles in blood agar for 24 h at 37 °C allowed the isolation of monomorphic colonies with alpha haemolysis. Microscopic examination after Gram staining showed monomorphic Gram-positive cocci. Isolated bacteria were catalase-negative and oxidase-negative. We used the BD Phoenix 100 system and BD Phoenix Gram Positive Panel (both Becton Dickinson Microbiology Systems) for phenotypic identification of the isolated colonies. The identified bacterium was *LLac* with 99.99 % confidence. The main reactions that distinguished our isolated bacterium from all other catalase-negative Gram-positive cocci were negative pyrrolidonyl-arylamidase (pyrrolidonyl aminopeptidase) and negative leucine aminopeptidase (leucine-arylamidase) [13].

As there are no standard methods or interpretation criteria of antimicrobial susceptibilities established for *LLac*, we adopted the breakpoints of the streptococci based on the European Committee on Antimicrobial Susceptibility Testing (EUCAST) [14]. The method used was Muller–Hinton agar diffusion. Inhibition diameters were based on the streptococcal antibiogram. Our strain was resistant to vancomycin, teicoplanin, rifampin and sulfamethoxazole-trimethoprim. *LLac* was susceptible to β-lactams, gentamicin, streptomycin, azithromycin, clarithromycin, lincomycin, clindamycin and erythromycin. After bacteriological examination and based on the results, initial antibiotic therapy was maintained for 10 days, including 6 days of ceftriaxone (50 mg kg^–1^ 24 h^–1^), followed by 4 days of oral amoxicillin (50 mg kg^–1^ 24 h^–1^). In addition, this regimen was combined with gentamicin (5 mg kg^–1^ 24 h^–1^) for 3 days. Her course was marked by apyrexia at day 2, and decreased C-reactive protein to 26 mg l^−1^. Our patient was discharged on the sixth day and died 1 year later from a haemorrhagic syndrome related to her disease.

## Discussion

*LLac* is a Gram-positive bacterium known by several features including spherical or lenticular form [15]. It is non-sporulated, capsulated, immobile, and with negative oxidase and catalase activities. It is also a facultative aero-anaerobic and heterofermentative lactic acid bacterium [15]. *LLac* is used in the food industry, especially in dairies and sugar factories, which explains its carriage in the oral cavity and the digestive tract of humans and animals [16–18]. *LLac* grows easily on columbia blood agar, but it is difficult to identify because of its rarity, and it can be morphologically confused with *Enterococcus* sp. or *Streptococcus* sp. in clinical microbiology laboratories where bacterial identification is limited to classical biochemical techniques [15]. It shares common characteristics with alpha-haemolytic or non-haemolytic *Streptococcus* sp. or *Enterococcus* sp. [19]. In addition, the increasing existence of vancomycin-resistant strains of enterococci may lead to confusion with other bacteria that are naturally resistant to these glycopeptides [19–22].

The difference between different genera of the family *Leuconostocacae*, essentially between *Leuconostoc* sp. and *Weissella* sp., can only be determined with molecular biology techniques, particularly metagenomic sequencing using the 16S rRNA marker [23, 24]. In fact, *Weissella* sp. belongs to the family *Leuconostocacae* and it shares the same biochemical characteristics with *Leuconostoc* spp., and it is also known as an opportunistic pathogen [23, 24]. In our case report, infection by *Weissella* sp. could not be excluded.

Since the 1990s, various types of infections have been described confirming the potential human pathogenicity of *LLac* [25]. However, its modes of transmission and pathophysiology remain unknown [26]. Described factors associated with infection include compromised immunity, previous antibiotic therapy (particularly vancomycin), parenteral nutrition, central venous catheters, surgery, liver failure, chronic renal failure, haemodialysis and extensive burns [12, 19, 27, 28]. Yet, *LLac*-associated infections have also been documented in healthy patients [29]. To our knowledge, we report the first case of *LLac* bacteraemia associated with osteopetrosis (Table 2). Osteopetrosis (‘marble bone disease’) encompasses a group of rare inherited autosomal metabolic bone diseases. One of the consequences of this fatal disease is pancytopenia [30]. There is currently no curative medical treatment for osteopetrosis [30]. Management of this disease is essentially supportive and aims to ensure multidisciplinary monitoring and symptomatic management of complications, and a supportive management of associated infections [31]. In our patient, pancytopenia, as a state of immunodeficiency, increased the risk of *LLac* bacteraemia. Another factor that might be associated with this bacteraemia is the multiple transfusion therapy protocol used to treat bone marrow insufficiency. The starting point could be the cutaneous route, either in the patient or the paramedical staff, but it could also be digestive as this is the reservoir of the bacterium [3]. The lack of clinical data on our proposal did not allow us to confirm with certainty the portal of entry of the bacteria in the blood nor the pathogenic mechanism of bacteraemia.

**Table 2.** Cases of *Leuconostoc lactis* infection reported in the literature

Author (year)	Country	Age	Gender	Specimen examined	Risk factors	Antibiotic therapy	Outcome
Azghar *et al*. (our case)	Morocco	4 years	Female	Blood	Pancytopenia Iterative transfusion Parenteral nutrition	Ceftriaxone, aminosid, amoxicillin	Cured
Yang *et al*. [9]	China	83 years	Female	Blood	Vancomycin therapy prior to infection Parenteral nutrition Central venous catheter	Linezolid	Death
Swain *et al*. [32]	India	62 years	Male	Blood	Diabetes Hypertension Myelofibrosis	None	Death before starting treatment
Deng *et al*. [8]	China	45 years	Male	Blood	Immunosuppressive therapy	Linezolid	Cured
Shin *et al*. [7]	South Korea	59 years	Female	Blood	Amyloidosis secondary to rheumatoid arthritis and tuberculosis, arthritis Vancomycin therapy prior to infection	Piperacillin and tazobactam	Cured
Koçak *et al*. [11]	Turkey	7 months	Male	Catheter Pus (skin) Blood	Central venous catheter Vancomycin therapy prior to infection	Cefotaxime	Cured
Deye *et al*. [10]	USA	50 years	Male	Cerebrospinal fluid	Vancomycin therapy prior to infection	Gatiﬂoxacin then penicillin G	Cured
Eleni *et al*. [33]	Greece	52 years	Male	Pus (liver)	Diabetes	Imipenem, netilmicin	Cure
Carapetis *et al*. [34]	Australia	Newborn	Female	Blood	Small bowel resection Parenteral nutrition Nasogastric tube Central venous catheter Vancomycin therapy prior to infection	Amoxicillin	Cured
Carapetis *et al*. [34]	Australia	4 years	Female	Blood	Gastrostomy Parenteral nutrition Vancomycin therapy prior to infection	Amoxicillin	Cured

After treating our patient, her course was marked by clinical and biological improvements and the patient was discharged 10 days after admission. To date, there are no recommendations for the treatment of patients infected with *LLac*. Published cases of *LLac* infection showed variability in the antibiotics used (Table 2). For example, penicillin, clindamycin, linezolid, macrolides, aminoglycosides, cephalosporins and tetracyclines were all tested [7, 8, 11]. Susceptibility to these or other antibiotics was not specified. The reported cases of death showed that this bacterial infection may be fatal [9, 32]. However, this was not the case for our patient. In fact, the combination of ceftriaxone and aminoglycosides led to a successful cure.

In summary, we discuss a case of a fragile patient who developed bacteraemia associated with low-pathogenicity or non-pathogenic but opportunistic bacteria. *LLac* is naturally resistant to vancomycin. This is an option for treating infections, but new drugs against Gram-positive bacteria are available (e.g. linezolid). Study of the sensitivity of *LLac* to antibiotics and the therapeutic protocol has not yet been standardized. Particularly noteworthy is the management of Gram-positive bacterial infections resistant to vancomycin, where clinicians must consider the presence of naturally vancomycin-resistant bacteria to avert severe consequences, especially with rare bacteria such as *LLac*. Additionally, the microbiology laboratory should assess susceptibility to alternative antibiotics to avoid therapeutic failures. The formulation of specific recommendations for such cases is eagerly anticipated to ensure effective treatment.

## Conclusion

We report a case of a compromised patient who developed bacteraemia with *LLac*. This species is known as a low-pathogenicity or non-pathogenic but opportunistic bacterium. The species must be determined to provide rapid effective therapy and improve clinical outcomes for these individuals.
